# Use of sofosbuvir based regimen in patients with end-stage renal disease and chronic hepatitis C; an open label, non-randomized, single arm, single center study from Pakistan 

**Published:** 2020

**Authors:** Rajesh Mandhwani, Farina M. Hanif, Ghulamullah Lail, Nasir Hassan Luck, Muhammad Ali Khalid, Muhammad Manzoor ul Haque, Syed Mudassir Laeeq, Tahir Aziz

**Affiliations:** 1 *Department of Hepatogastroenterology, Sindh Institute of Urology and Transplantation, Karachi, Pakistan. *; 2 *Department of Nephrology and Transplant Sciences, Sindh Institute of Urology and Transplantation, Karachi, Pakistan *

**Keywords:** End-stage renal disease, hemodialysis, Hepatitis C virus, Interferon, Sofosbuvir, Sustained virological response

## Abstract

**Aim:**

we aimed to determine the virological response and safety of Sofosbuvir-based direct-acting antiviral agents (DAAs) in chronic hepatitis C (CHC) patients on long-term hemodialysis (HD).

**Background:**

With the advent of interferon-free DAAs, the treatment of CHC has been revolutionized. Pakistan is among the countries where novel sofosbuvir (SOF)-free antiviral agents are not available.

**Methods:**

This non-randomized, single-arm, open-label study enrolled all HD patients with chronic HCV infection after informed consent. They were treated with SOF in combination with Ribavirin (RBV) with either interferon (IFN group) or daclatasvir (DAC group), with the virological response assessed according to standard guidelines. Data were analyzed using SPSS version 20.00.

**Results:**

Out of 133 patients, the majority (72.9%) were males with the mean age of 31.92 ± 9.88 years. Most patients (50.3%) had HCV genotype (GN) 1, followed by GN 3 in 42.9%, 4 in 1.48% and 2 in 0.7%, while mix GN was documented in 6 (4.4%) patients. Among these, 60 (45.1 %) patients received standard SOF, IFN, and RBV (IFN group) and 73 (54.9 %) received SOF, DAC and RBV (DAC group). End of treatment and sustained virological response at 12 weeks post-treatment were achieved in 133 (100%) and 129 (97 %) patients, respectively. The adverse effects were anemia in 58 (43.6 %) patients and elevated alanine transaminases in 11 (8.1%) patients.

**Conclusion:**

SOF in combination with either IFN or DAC is an equally efficacious and effective treatment regimen for patients on maintenance HD, especially in resource-poor countries.

## Introduction

 Patients with end-stage renal disease (ESRD) are considered to be at increased risk of acquiring hepatitis C virus (HCV) infection (1). Worldwide, in ESRD patients, its prevalence ranges from 6% to 60%, while in Pakistan, it is between 16.4% and 68% (2, 3). The ideal treatment of ESRD patients is renal transplantation. Nevertheless, concomitant HCV infection increases the risk of post-transplant graft rejection, proteinuria, infection, diabetes, and fibrosing cholestatic hepatitis (2, 4). Thus, prior HCV eradication improves outcomes in this subset of the patient population. 

the treatment of HCV infection has evolved from interferon (IFN) to direct-acting antiviral agents (DAAs). Nonetheless, non-availability, higher cost and lack of recommendations hamper the use of these novel agents in dialysis-dependent patients. Although it is preferable to use sofosbuvir (SOF)-free regimen in patients with severe renal impairment, HCV infection can be treated with SOF-based DAAs, if novel agents are not available (5, 6). At the time of the study, only SOF and Daclatasvir (DAC) were available in Pakistan, so we had very limited choices to treat hemodialysis (HD) patients with chronic HCV infection. Furthermore, our previous experience of SOF-based HCV treatment in renal transplant recipients (7), case reports, and published data of DAAs’ effectiveness in few dialysis patients encouraged us to use these agents in our HD population (8-10).

Accordingly, we aimed to evaluate the efficacy and safety profile of SOF-based DAAs in HD patients with chronic HCV infection. 

## Methods

This open-label, non-randomized, single arm, single center study, included all ESRD patients on maintenance HD with chronic HCV infection during the period from Jan 2016 to June 2018, who consented to be treated with DAAs. Inclusion criteria were: all consecutive adult patients on long-term maintenance HD of either gender who tested positive for HCV testing, who were treatment naïve and were willing to undergo renal transplantation with suitable living related donor available in the family. Exclusion criteria were: patients not willing to undergo transplantation or the presence of established cirrhosis. Any treatment-experienced patients were also excluded. Written informed consent was taken after explaining that data regarding the use of SOF and DAC in dialysis patients is sparse. Patients were allowed to withdraw in case of any intolerable adverse effect or if they wished to discontinue DAAs therapy at any point. The Diagnosis of HCV infection was documented by HCV RNA through PCR and genotyping (Roche CobasTaqMan and Abbott Real-Time HCV). None of the patients underwent liver biopsy; cirrhosis was assessed clinically and radiologically with patients with cirrhosis excluded from the study.

Irrespective of genotype, all patients were initially prescribed standard IFN, 3 mIU, thrice weekly, combined with SOF, 400 mg (only DAA was available in Pakistan during the study period) once daily and ribavirin (RBV), 200 mg, twice weekly for 3 months (IFN group) (11).A few months later, DAC became available in Pakistan, so all other new patients were given DAC, 60 mg and SOF 400 mg once daily along with RBV 200 mg twice weekly for three months (DAC group). For the initial 4 weeks, the patients were followed-up fortnightly along with biochemical tests; later, they were followed up monthly or given clinical indications. 

HCV RNA by PCR was checked at 4 and 12 weeks to document rapid virological response (RVR) and end-of-treatment response (ETR). In patients who failed to achieve RVR, the treatment was extended for a further 3 months. After completion of treatment, the sustained virological response was checked at 12 weeks (SVR12).

This study was approved by the Institutional Ethics Committee and was carried out in accordance with the tenets of the Declaration of Helsinki.

**Statistical analyses**

Data were analyzed using SPSS Statistics software (SPSS: An IBM Company, version 20.0, IBM Corporation, Armonk, NY, USA). The results were presented as means ± SD for quantitative data or as numbers with percentages for qualitative data. A comparison of quantitative variables with pre- and posttreatment were analyzed using the unpaired Student *t-*test. A *p-*value of <0.05 was considered statistically significant. 

## Results

Out of 151 patients initially enrolled, 18 did not turn up for the start of HCV treatment and thus were not included in the analysis. Thus, 133 patients were analyzed with the majority being males (72.9%) with the mean age of 31.92 ± 9.8 years. None of our patients underwent peritoneal dialysis. The duration of acquiring HCV infection to treatment initiation could not be assessed as patients were referred only if a suitable live-related renal donor was available and HCV treatment was indicated. The majority of patients had GN 1 (50.3%), followed by GN 3 in 42.9%, GN 4 in 1.48% and GN 2 in 0.7%, while mix GN was documented in 6 (4.4 %) patients (Table 1). 

**Figure 1:**
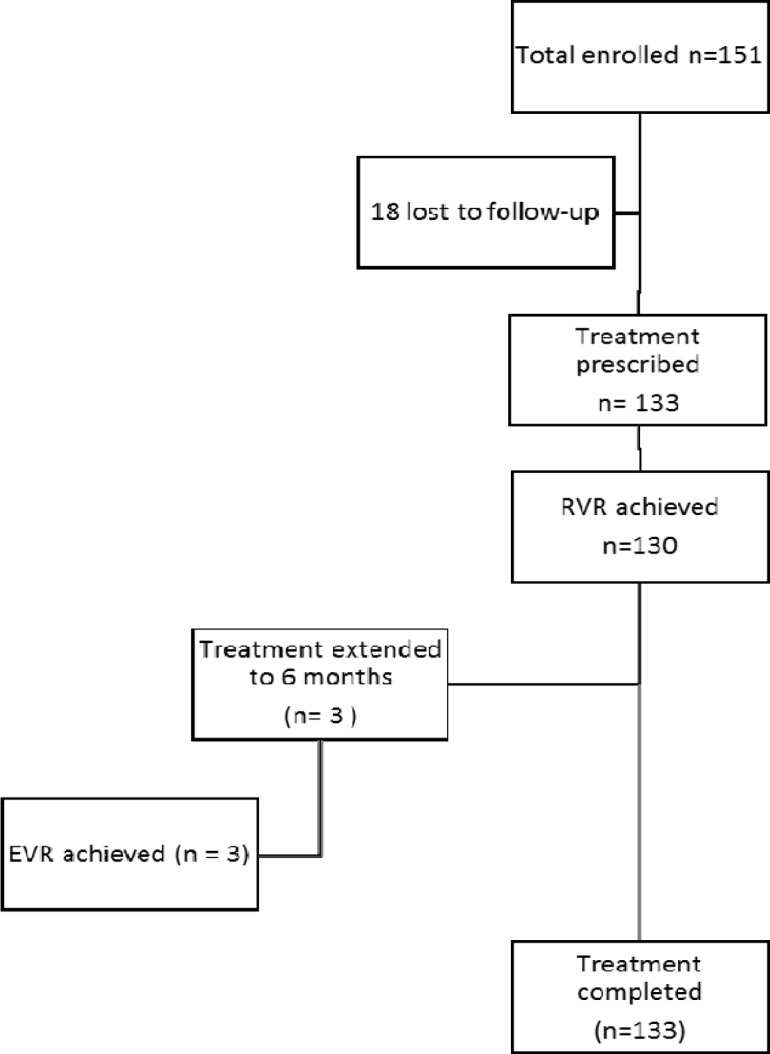
Flow chart of the study population

In total, 60 patients (45.1 %) received standard IFN, SOF and RBV (IFN group) while 73 (54.9%) received SOF, DAC and RBV (DAC group). RVR was achieved in 130 (97.7%) patients; in the remaining three patients, the treatment was extended to 24 weeks and all had undetected HCV PCR at 12 weeks of treatment, as displayed in Figure 1.

**Table 1:** Baseline characteristics of the study population (n = 133)

Age in years (mean ± SD)	31.92 ± 9.88
Gender, Male/Female, n (%)	97 / 36 (72.9 / 27.1)
Duration of Hemodialysis (months)	26.49 ± 28.11
Genotype: n (%)	
1 2 3 4 Mix (1 & 2) Mix ( 1 & 3 )	67 (50.3 %) 1 (0.7%) 57 (42.9% ) 2 (1.48%) 1 (0.7%) 5 (3.7%)

EVR: early virological response, RVR: rapid virological response

Overall, treatment was completed by all 133 patients and ETR was achieved in all (100%). SVR12 was achieved in 129 (96.9 %) patients. In the remaining four patients, 2 had documented virological relapse while the other two had new genotypes as compared to nadir, representing new infections. Two of these belonged to GN 1 and 2 to GN 3. Three of these were in DAC group, while one in IFN group and all four received a 3-month treatment. The drug regimens and the results of virological responses are reported in Table 2. 

The statistically significant decline in hemoglobin, platelet count, and liver function tests were noted, while albumin level improved with treatment with DAAs (Table 3). 

**Table 2:** Drug regimen and virological Response of the study population

	IFN Group (SOF+IFN+RBV) n=60	DAC Group (SOF+DAC+RBV) n=73
RVR	59 (98.3%)	71 (97.3%)
ETR	56 (100 %)	73 (100 %)
SVR 12	59 (98.3%)	70 (95.9%)
Non-responders	1 (1.6%)	3 (4.1%)
3-months treatment	59 (98.3%)	71 (97.3%)
6-months treatment	1 (%)	2 (2.7%)

Dac: Daclatasvir, ETR: End of treatment response, IFN: interferon, RBV: Ribavirin RVR: Rapid Virological Response, SOF: Sofosbuvir, SVR12 Sustained Virological Response at 12 weeks

The adverse effects noticed included anemia, elevated alanine transaminases, and seizure. Anemia was noticed in 58 (43 %) patients, among whom, 31 (23 %) required blood transfusion (20 in IFN group and 11 in DAC group) while RBV dose was reduced in remaining 27 (20.3%) patients (10 in IFN group and 17 in DAC group). Seizure was documented in 

**Table 3:** Laboratory parameters before and one week after completion of the treatment (n = 133)

	Pre treatment	Post treatment	*p-Value*
Hemoglobin, g/dl	9.71±1.4	9.16 ±1.5	< 0.001
Total leukocyte count, x 10^9^/L	7.36 ±2.17	7.37 ± 4.21	0.67
Platelet count, x 10^9^/L	256.06 ± 98.93	226.52±94.90	< 0.001
Total bilirubin, mg/dl	0.72 ± 0.28	0.70±0.25	0.015
Alanine aminotransferase, U/L	43.69±36.33	22.63 ± 21.30	< 0.001
Aspartate aminotransferase, U/L	39.70 ±27.39	25.11±19.41	< 0.001
Gamma-glutamyltransferase , U/L	73.53± 69.21	42.34±33.49	< 0.001
Albumin, mg/dl	3.43±0.47	3.60±0.43	< 0.001
International normalized ratio	1.12±0.13	1.17±0.15	0.196

p <0.05 was considered statistically significant

only one patient on IFN regimen. Alanine transaminase was elevated during treatment in 11 (8.1 %) patients in DAC group and 5 patients in IFN group.

## Discussion

To the best of our knowledge, we report the largest number of HD patients treated for HCV infection with SOF-based DAAs. Considering the high prevalence of HCV infection in different dialysis centers of Pakistan, i.e. 16.4 to 68 % (3), studies on the efficacy and safety of these DAAs can bring significant improvements in the management of this special patients’ group especially in countries with limited drug availability.

HD is a risk factor for the acquisition of HCV infection which usually progresses to chronic infection (11-13). Due to lack of precautionary measures, HD patients have a higher prevalence of HCV infection as compared to the general population (12, 14, 15). Anti-HCV positive HD patients have both a lower survival rate and a higher incidence of hepatocellular carcinoma and cirrhosis (16, 17). Thus, withholding HCV treatment until renal transplant may lead to progression of liver disease (18). In our center, only the HD patients with a suitable live-related donor in the family and willing to undergo transplantation were offered anti-HCV treatment. 

Agarwal et al. (2), conducted a study on HD patients of India with HCV infection with the majority being infected with GN 1. Likewise, GN 1 was most commonly observed in our study population, i.e., in 61 (51.3%) patients. These findings are in contrast to the report that the most common GN in Pakistan and India is GN 3. Messina et al. (19) hypothesized that the global distribution of GN 1 may be secondary to dissemination of contaminated blood and blood products prior to the discovery of HCV, while the distribution of GN 3 can be due to population migration. None of our patients had any history of travel abroad. However, being a single-center study, of a specific group, the results cannot be extrapolated to the entire population.

In our study population, 6 patients had a mixed genotype infection. The treatment of mixed genotype HCV infection with DAAs is sparse (5); nevertheless, we documented overall SVR12 of 96.9% in our study population which included 6 patients with a mixed GN.

The treatment of HCV in HD patients has been reformed from IFN to the advent of DAAs. Until recently, DAAs were not recommended as a treatment option in HD population. It is advisable to use SOF-based anti-viral agents when other recommended agents are not available (5, 6). The hindrance in utilizing SOF in renal insufficient patients is associated with its renal excretion pathway. Moreover, there is no recommended dose in patients with ESRD (5, 6, 8). Considering higher accumulation of drug and its metabolites, researchers have observed variable response with SOF dose modification (20).

Bhamidimarri et al. (21) documented 91% vs. 75% SVR12 in 15 patients on daily 200 mg vs. 400 mg alternate day SOF, respectively. In the above-mentioned study, out of a total 15 patients, 12 were HD-dependent. Agarwal et al. (2) documented virological relapse in all 3 patients treated with SOF, 400 mg, on alternate days. Desnoyer et al. (22) noted the non-accumulation of SOF and its metabolite in 12 HD patients. They also documented virological relapse in two patients out of 5, treated with three times per week SOF regimen. We prescribed the full dose of SOF, i.e., 400 mg per day in all our patients and virological relapse was documented in two (1.5%) patients.

Agarwal et al. (2) reported 5.2% SVR12 in 62 HD patients treated with different doses and a combination of SOF. Aggarwal et al. (9) documented lower SVR12 i.e., 86.7 % in 15 dialysis patients which can be attributed to eight (57%) treatment-experienced patients. A meta-analysis of 11 studies, based on DAA treatment of HCV patients with stage 4-5 chronic kidney disease, demonstrated pooled SVR12 of 89.4% in patients with SOF-based DAAs and 94.7% in patients with non-SOF based DAAs (8). The meta-analysis of 9 studies found pooled RVR of 88.0%. All our patients were treatment-naïve and we documented RVR and SVR12 of 97.7% and 96.9%, respectively, on treatment with SOF-based DAAs.

In a real-world cohort, HCV TARGET study (10) included 1789 HCV patients with renal insufficiency. He documented a higher frequency of anemia irrespective of RBV, deteriorating renal functions and more adverse effects in 73 patients with estimated GFR≤ 45 ml/min/1.73 m². However, dialysis-dependent patients were only 5 out of 73. AASLD (5) and EASL (6) recommended close monitoring of patients with renal dysfunctions treated with SOF-based regimen due to the risk of renal deterioration. On the other hand, our study population consisted of dialysis-dependent patients. 

The adverse effects observed in our study population included anemia, elevated alanine transaminases (ALT), and seizure. Agarwal et al. (2) demonstrated the higher requirement of erythropoietin in 56 % of the study population. Aggarwal et al. (9) also observed anemia in one patient with RBV, with the patients also showing concomitant sepsis. In our study, anemia was noticed in 58 (43%) patients who were treated with RBV, more commonly in IFN group than in DAC group (30 in IFN group and 28 in DAC group); RBV dose modification and blood transfusion were undertaken depending on the degree of drop of hemoglobin from the baseline. A drop of >1 g/dl in hemoglobin was treated as significant anemia. Blood transfusion was undertaken in 31 patients when hemoglobin dropped below 8.5 g/dl. A significant fall in hemoglobin above this cut-off (1 g/dl or above) was treated by reducing RBV dose (27 patients) to once weekly. Liver enzymes were raised in 11 patients (6 in DAC group and 5 in IFN group). The rises in serum ALT ranged within 80-152 U/l. The treatment was held for one week in two patients with serum ALTs of 103 and 152 U/l. The treatment was resumed once ALT was normalized. In the remaining 9 patients, treatment was not discontinued. 

Although we documented 96.9 % SVR12 in 133 dialysis-dependent patients, our study had some limitations. First of all, the patients were treated with standard IFN as it was easily available and one of the recommended treatment options in our study population (11). A randomized control trial comparing Pegylated IFN and standard IFN in HD patients documented no statistically significant difference in virological relapse, SVR, mortality, side effects, and treatment cessation (23). Secondly, anemia was most frequently seen in our study, which can be attributed to the use of RBV. We did not use RBV-free SOF and DAC combination as effectiveness of this regimen was not known. Further, SVR-12 was not achieved in 4 patients. Two of these had new HCV infection while virological relapse was noted in the other two. In later patients, we did not perform resistance or phylogenetic analysis to differentiate true virological relapse or new HCV infection.

Considering the non-availability and high cost of the latest DAAs in developing countries, our experience will help reduce the hesitancy in using SOF-based DAAs in ESRD patients. Thus, we conclude that SOF in combination with IFN or DAC can be prescribed in dialysis-dependent patients not only in genotypes 1 and 3 but also with mix genotype HCV infection.

## Conflict of interests

The authors declare that they have no conflict of interest.
